# Models and Biomarkers for Local Response Prediction in Early-Stage and Oligometastatic Non-small Cell Lung Cancer Patients Treated With Stereotactic Body Radiation Therapy Using Machine Learning

**DOI:** 10.7759/cureus.75819

**Published:** 2024-12-16

**Authors:** Gemini Ramasamy, Thierry Muanza, Goulnar Kasymjanova, Jason Agulnik

**Affiliations:** 1 Department of Experimental Medicine, McGill University, Montreal, CAN; 2 Division of Radiation Oncology, Sir Mortimer B. Davis Jewish General Hospital, McGill University, Montreal, CAN; 3 Department of Medicine and Pulmonary Oncology, Sir Mortimer B. Davis Jewish General Hospital, McGill University, Montreal, CAN; 4 Anna and Peter Brojde Lung Cancer Center, Sir Mortimer B. Davis Jewish General Hospital, McGill University, Montreal, CAN

**Keywords:** local response, non-small cell lung cancer, predictive, radiomics, stereotactic body radiation therapy

## Abstract

Background

A minority of patients receiving stereotactic body radiation therapy (SBRT) for non-small cell lung cancer (NSCLC) are not good responders. Radiomic features can be used to generate predictive algorithms and biomarkers that can determine treatment outcomes and stratify patients to their therapeutic options. This study investigated and attempted to validate the radiomic and clinical features obtained from early-stage and oligometastatic NSCLC patients who underwent SBRT, to predict local response.

Methodology

A single-institution, Institutional Review Board (IRB)-approved retrospective review was conducted on adult patients with early-stage and oligometastatic SBRT-treated NSCLC at the Jewish General Hospital. The study included 98 patients (82 with early-stage NSCLC and 16 with oligometastatic disease), with a median age of 76 years and a male-to-female ratio of 46:52. A total of 116 lesions were treated with SBRT between 2009 and 2022. Radiomics features (*n* = 107) were extracted from CT planning scans using PyRadiomics, and clinical data were collected for all 98 patients. Local response was assessed according to Response Evaluation Criteria In Solid Tumors (RECIST 1.1) criteria. Classification models, including support vector machines, random forests, adaptive boosting, and multi-layer perceptrons (MLPs), were used. Models were trained using a fivefold cross-validation scheme. Their performances were measured with receiver operating characteristic plots on the validation folds. Using the importance of the permutation feature, predictive biomarkers were identified.

Results

The most predictive model, incorporating all patients and using an MLP classifier with Adaptive Synthetic (ADASYN) sampling, a combined-input approach, and a radiomic filter, achieved an area under the curve (AUC) of 0.94 ± 0.05. When oligometastatic patients were omitted, the best model (AUC 0.95 ± 0.06) was also predictive, using a support vector classification (SVC) radial basis function (RBF) classifier, ADASYN sampling, and a clinical-based input. Treatment site and performance status, along with radiomic features such as first-order root-mean-squared-intensity, first-order skewness, and gray-level nonuniformity, were found to be predictive biomarkers.

Conclusions

The predictive models generated and the biomarkers identified could be used in clinical decision support systems for SBRT-treated NSCLC patients. Additionally, treatment site, performance status, and radiomic features were the most predictive variables.

## Introduction

Lung cancer is one of the most common types of cancer in humans [1]. It is the leading cause of cancer-related deaths in the world [2]. Mainly caused by smoking tobacco and other carcinogens [3], lung tumors predominantly develop into non-small cell lung cancer (NSCLC) [1].

When treating NSCLC patients, many treatments are available depending on the cancer progression stage. For early-stage NSCLC patients, surgical resection is the standard treatment [4]. However, due to medical complications, comorbidities, or refusal of surgery, stereotactic body radiation therapy (SBRT) is recognized as the standard alternative treatment for medically inoperable patients [4-6].

Stating the problem

While SBRT results in effective local control of surgically inoperable early-stage and oligometastatic NSCLC tumors, a small proportion of tumors do not respond well to the treatment [7,8]. Also, some of these tumors suffer from local recurrence [7,8]. Indeed, in a prospective trial, SBRT was used on medically inoperable, early-stage, NSCLC tumors (Radiation Therapy Oncology Group [RTOG] 0236 trial) [7]. After three years, primary tumor local control was observed, with a rate of 97.6%. Consequently, about 2.4% of patients were poor responders to the treatment [7].

For patients who do not respond well to SBRT, modifications can be applied to the treatment regimen. For example, dose escalation - when paired with SBRT - was shown to improve local control for early-stage NSCLC patients who cannot receive surgical resection [8,9,10].

Hypothesis

In this study, we hypothesized that pretreatment data have predictive potential, specifically in the form of biomarkers [11]. Medical images, such as computed tomography scans, may contain a wealth of pathophysiological information, which could be extracted as agnostic, radiomic features and used for clinical decision support systems [11]. Indeed, these data may be utilized to generate predictive, machine-learning-based algorithms and biomarkers, which may predict treatment outcomes.

Rationale

Incorporating predictive models and biomarkers as support systems would help physicians to better stratify patients at risk of treatment failure. Such tools would be important because patients at risk would be in poorer health and additional treatment morbidity would negatively impact the patients’ quality of life [8]. While physicians use clinical variables, semantic, and radiological features to make treatment decisions, radiomic features hold highly predictive and nuanced information for every patient and tumor. Such features would lead to personalized and tangible modifications regarding SBRT workflows [12]. Indeed, using predictive, radiomic features to identify patients with high, local failure risk would facilitate the decision to give a more aggressive treatment, such as modified treatment volumes, dose escalations, and closer monitoring of patients [12]. Using predictive models and biomarkers in clinical settings would enable risk-adapted therapeutic protocols.

Objectives

In this study, we conducted a retrospective review of early-stage and oligometastatic NSCLC patients treated with SBRT, using medical records from the Jewish General Hospital (JGH). We focused on assessing treatment response and collecting pre-SBRT radiomics and clinical data. The objective was to use these data to train machine learning algorithms to predict patients’ local response after treatment. Also, using a multi-modality approach, we compared the performance of radiomics-based models, clinical-based models, and combined-based models. Our second objective was to identify and validate important biomarking features used by the models. Establishing the applicability of biomarkers to different patient cohorts could inspire confidence in other researchers to incorporate these biomarkers in their studies.

Sections from this study were previously presented as meeting abstracts and posters at the 2024 Canadian Association of Radiation Oncology (CARO) Annual Scientific Meeting on September 12, 2024, and at the 2024 American Society for Radiation Oncology (ASTRO) Annual Scientific Meeting on October 1, 2024.

## Materials and methods

Retrospective review

To establish our population dataset, we conducted an IRB-approved retrospective review of lung cancer patients treated at JGH from 2009 to 2022. The study was approved by the Research Ethics Board of CIUSSS West-Central Montreal (IRB approval number: 2019-1488). We applied our inclusion/exclusion criteria to identify eligible patients for our study. Using the 8th edition of the TNM staging system, we selected 98 adult patients (18 years or older) with early-stage (stage I or II, without nodal involvement) or stage IV oligometastatic NSCLC, who were treated with SBRT. In this study, oligometastatic disease featured a limited spread of microscopic metastases in the lungs of NSCLC patients. Additionally, each patient had their planning CT scans available, along with at least one follow-up CT scan or chart. Among the patients, 116 tumor lesions that were treated were identified. For each patient, clinical information was collected and used as clinical variables in our machine-learning dataset. Clinical variables included age at treatment, sex, smoking status, smoking pack-years, number of SBRT fractions, SBRT dose (Gy), dosimetry parameters (such as V12 for the lung, a dose-volume histogram parameter), performance score, TNM staging, and treatment site. Other useful information was also included in our retrospective study, such as the number of lesions per patient, death event/last follow-up date, and the number of months between the start of SBRT and death event/last follow-up date. For each clinical feature mentioned above, at least 80% of lesions had available data. Any clinical variable with more than 20% of missing data was not retained in our study. CT scans, most of which had a size of 512 x 512 x 112 voxel units, were exported from the JGH database. Additionally, tumor contouring (including internal target volume [ITV] and planning target volume [PTV]) was performed by trained physicians using the CT planning scans.

Population dataset: Early-stage and oligometastatic NSCLC patients

In this study, both early-stage and oligometastatic NSCLC patients were used to create predictive models. Even though the extent of the disease and the clinical outcomes are different when comparing early-stage and oligometastatic NSCLC cases, there are grounds to investigate them together.

First, early-stage NSCLC tumors and NSCLC oligometastases are treated with SBRT to achieve the same goal; SBRT delivers an ablative radiation dose to enhance local control of the targeted tumors. This specific objective can be evaluated using local control as the treatment outcome. Regardless that the probabilities for a complete or partial local response may be different when contrasting early-stage and oligometastatic NSCLC, SBRT is still suggested for the local control of small tumors.

Second, the inclusion of early-stage and oligometastatic NSCLC patients in one study increases the number of samples available when training and testing predictive models. Indeed, when training and testing predictive models with machine learning, the amount of original, high-quality samples is important; the SBRT-targeted tumors in early-stage and oligometastatic NSCLC patients share enough common characteristics to study them together, in the context of predictive algorithms. Furthermore, this study trained models to predict the response of only early-stage cases, as well as the response of early-stage and oligometastatic cases, combined. This study design allowed us to compare two types of sample sets. However, models trained with only oligometastatic cases were not created in this study because the number of oligometastatic cases was low.

For all these reasons, early-stage and oligometastatic, SBRT-treated, NSCLC cases were included in this study.

Response assessment

The local response was assessed manually, using the Response Evaluation Criteria In Solid Tumors (RECIST 1.1) [13]. This method evaluated the local response at a specific timepoint, by factoring in the volume progression of the tumor since the treatment was given. This assessment was done by comparing the last CT scan before treatment, the treatment planning CT scan, with the last follow-up CT scan. Following RECIST, the local response was categorized as complete response (CR), partial response (PR), stable disease (SD), and progressive disease (PD) [13].

When discussing the response of diseases to treatment in the context of predictive algorithms, it is often favored to have the response assessed as positive or negative. In the case of local response, because there are four distinct classes, the categories were grouped in a manner that reflects a binary classification. CR and PR were grouped as positive responses, and SD and PD were grouped as negative responses. Trained with this classification, the algorithm predicted whether or not the ablative nature of SBRT was effective and whether or not SBRT eliminated a significant portion of preexisting malignant cells.

Data anonymization

This study obtained approval from the Institutional Review Board (IRB) for the collection and analysis of stages I, II, and IV (specifically oligometastatic) NSCLC patients treated with SBRT at the JGH - affiliated with McGill University - between 2009 and 2022. After the scans were exported and the clinical data were collected, as per IRB regulations, they were anonymized to remove any identifiable information about the patients. The patients' data were anonymized with a code assigned by the principal investigator and their team. The data were then stored separately and securely, in accordance with IRB regulations.

CT scans preprocessing

Before extracting radiomic features, the CT scans underwent preprocessing steps, using PyRadiomics [14]. All preprocessing steps for CT scans were done individually and in isolation, and no data were shared between images. Preprocessing steps included image normalization, image/mask resampling, re-segmentation, and mask validation. First, normalization centered the image at the mean intensity value with its standard deviation, and it removes intensity values outliers using a 95% confidence interval by taking into account the entire CT image voxel intensities [14]. Second, image/mask resampling resampled the CT scan and the contouring mask to a specified voxel size. In our case, the resampled voxel size was set to 1 x 1 x 1 units, corresponding to the *x*, *y*, and *z* planes [15]. Third, re-segmentation adjusted the contouring mask to include voxel intensities within three standard deviations above and below the mean intensity, maintaining a 95% confidence interval [14,15]. Finally, mask validation verified whether the mask's contoured region of interest properly corresponded to the CT image's mask size, dimensions, spacing, direction, and origin [14]. These preprocessing steps were necessary to extract reproducible radiomic features [14].

Radiomic features extraction

CT scans contain multiple radiomic features. To extract these features, we used the Python-based PyRadiomics module [14]. As most of this Python-based module abides by the Transparent Reporting of a Multivariable Prediction Model for Individual Prognosis or Diagnosis (TRIPOD) statement, it was an ideal radiomics extractor for this project [16]. Before feature extraction, different filters were used to modify the CT image. The applied filters included original, wavelet, Laplacian of Gaussian (LoG), square, square root, logarithm, exponential, gradient, local binary pattern 2D (by-slice operation), and local binary pattern 3D (using spherical harmonics). Indeed, while we extracted features from the original scan, we also collected features from the derived, filtered versions of the scans [17]. With this module, 107 features were extracted from the three-dimensional region of interest of the CT scans. For this project, extracted, radiomic classes included shape-based 3D features, first-order features, and texture features. Specifically, texture features included sub-categories, such as gray-level co-occurrence matrix (GLCM), gray-level run length matrix (GLRLM), gray-level size zone matrix (GLSZM), neighboring gray-tone difference matrix (NGTDM), and gray-level dependence matrix (GLDM) [14,18].

Input arrays

As this project investigated the effect of using different types of features to make predictions about treatment, three input arrays were formatted: a radiomics-based array, a clinical-based array, and a combined array (clinical and radiomic features). Using these data arrays, we tested the performance of a multimodality approach.

Data imputation

While conducting the retrospective review, some patients did not have all clinical variables readily available. As most machine learning models do not deal well with missing values, we used data imputation to mitigate missing values in the clinical array. Indeed, we used a simple imputer, which replaced the missing values with the most frequent value for the specific feature [19]. The imputed variables included the performance score, the smoking status, the smoking pack years, and the V12 dosimetry parameter. Also, all imputed variables had 80% or more original values.

Data augmentation

To attain an improved balance between positive and negative responses/classes, we used the ADASYN sampling method to over-sample the class with the lowest number of sample lesions. This method helps improve the performance of the models by focusing on the samples that were difficult to classify.

Standard scaling

Before inputting data arrays in machine learning models, we standardized the values in a feature by scaling and centering the data [19,20]. Standard scaling occurred after the dataset was split into training and testing folds.

Machine learning algorithms for classification

For this project, we performed machine learning and classification tasks. We wanted the classifiers to properly identify which lesion would have a positive or negative response, based on their unique features. There were many supervised learning estimators, which could be used as classifiers. Considering the relatively small amount of sample lesions in our dataset (116 lesions), the support vector machine (SVM) classifier, with linear basis function or RBF kernel, and the random forest classifier were both good options to consider. It is important to note that our SVMs were used for classification; therefore, SVM classifiers were referred to as *support vector classification* (SVC) classifiers. Also, we tried more advanced options, such as the adaptive boosting (AdaBoost) classifier and the multi-layer perceptron (MLP) classifier.

Cross-validation

There are many advanced versions of the cross-validation method, but for this project, we used a stratified shuffle split cross-validation for training and testing our models [19,21]. For our purposes, fivefold was enough; 80% of the dataset trained the model, and 20% of the dataset was used for testing.

Receiver operating characteristic curve

To evaluate the predictive performance of models, receiver operating characteristic (ROC) curves can be computed using testing subsets [22]. ROC curves represent the progression of the true positive rate (sensitivity) versus the false positive rate (1-specificity), as the classification threshold between two classes explores the range of data points.

To quantify the performance of the model, the area under the curve (AUC) is calculated; the closer the AUC’s value is to 1, the more predictive a model is. An ROC curve was plotted for each cross-validation fold's testing set, and the average AUC of those curves was calculated at the end, thus giving the overall performance of the model [22,23].

Statistical significance

When comparing the performance of different model configurations with different inputs, the p-value of comparable models was calculated using a one-way analysis of variance (ANOVA) method, with a 95% confidence interval [24].

Permutation feature importance

Permutation feature importance was plotted as box plots for each feature to show the decrease in accuracy score. The larger the decrease in accuracy score was for a specific feature, the more important that feature was for that fitted model. Feature importance was calculated for each cross-validation fold's testing set, and the average feature importance was calculated at the end, thus resulting in a feature's overall importance for a specific fitted model [25].

## Results

Table 1 compares the performance of models for the prediction of local response, based on different configurations. For all respective classifiers, adding the ADASYN sampling method to the machine learning pipeline dramatically increased model performance. When all patients were included, the MLP model achieved the highest AUC (0.81 ± 0.1) among the radiomics-based models, while the random forest model had the highest AUC (0.85 ± 0.08) among the clinical-based models. Although, the combined-based, MLP model exceeded both single-modality models with an AUC of 0.87 ± 0.09. That said, a one-way ANOVA between the best models in each input category demonstrated no statistical difference (*P* = 0.58).

**Table 1:** Performance comparison of different models for local response prediction of early-stage and oligometastatic patients. ANOVA, analysis of variance; AUC, area under the curve; SD, standard deviation; ADASYN, adaptive synthetic; SVC, support vector classification; RBF, radial basis function; AdaBoost, adaptive boosting; MLP, multi-layer perceptron

Classifier	Data augmentation	Data input						One-way ANOVA
		Radiomics		Clinical		Combined		
		Mean (AUC)	SD (AUC)	Mean (AUC)	SD (AUC)	Mean (AUC)	SD (AUC)	*P*-value (alpha = 0.05)
SVC RBF, c1, ovo	No ADASYN	0.27	0.2	0.4	0.14	0.31	0.1	0.41
	ADASYN	0.73	0.13	0.84	0.15	0.85	0.08	0.27
SVC linear	No ADASYN	0.55	0.08	0.31	0.09	0.51	0.11	0.0036
	ADASYN	0.75	0.16	0.47	0.2	0.76	0.08	0.019
Random Forest Classifier, 55 estimators	No ADASYN	0.36	0.16	0.33	0.12	0.36	0.18	0.94
	ADASYN	0.81	0.15	0.85	0.08	0.83	0.12	0.87
AdaBoost, 300 estimators	No ADASYN	0.3	0.19	0.58	0.19	0.45	0.11	0.064
	ADASYN	0.76	0.05	0.77	0.07	0.81	0.04	0.34
MLP, sgd, alpha 0.001	No ADASYN	0.48	0.1	0.48	0.09	0.51	0.08	0.83
	ADASYN	0.81	0.1	0.8	0.11	0.87	0.09	0.51
ANOVA P-value comparison between best models		0.81	0.1	0.85	0.08	0.87	0.09	0.58

Although the performance of the combined-based model increased with the filtered radiomic features, it was not statistically significantly different (*P* = 0.063) compared to the radiomics-based and clinical-based models (Table 2).

**Table 2:** Adjusted performance comparison of highest performing models with different data inputs for local response prediction of early-stage and oligometastatic patients. ANOVA, analysis of variance; AUC, area under the curve; SD, standard deviation

Data input						One-way ANOVA
Radiomics		Clinical		Combined		
Mean (AUC)	SD (AUC)	Mean (AUC)	SD (AUC)	Mean (AUC)	SD (AUC)	*P*-value (alpha = 0.05)
0.81	0.1	0.85	0.08	0.94	0.05	0.063

**Figure 1:**
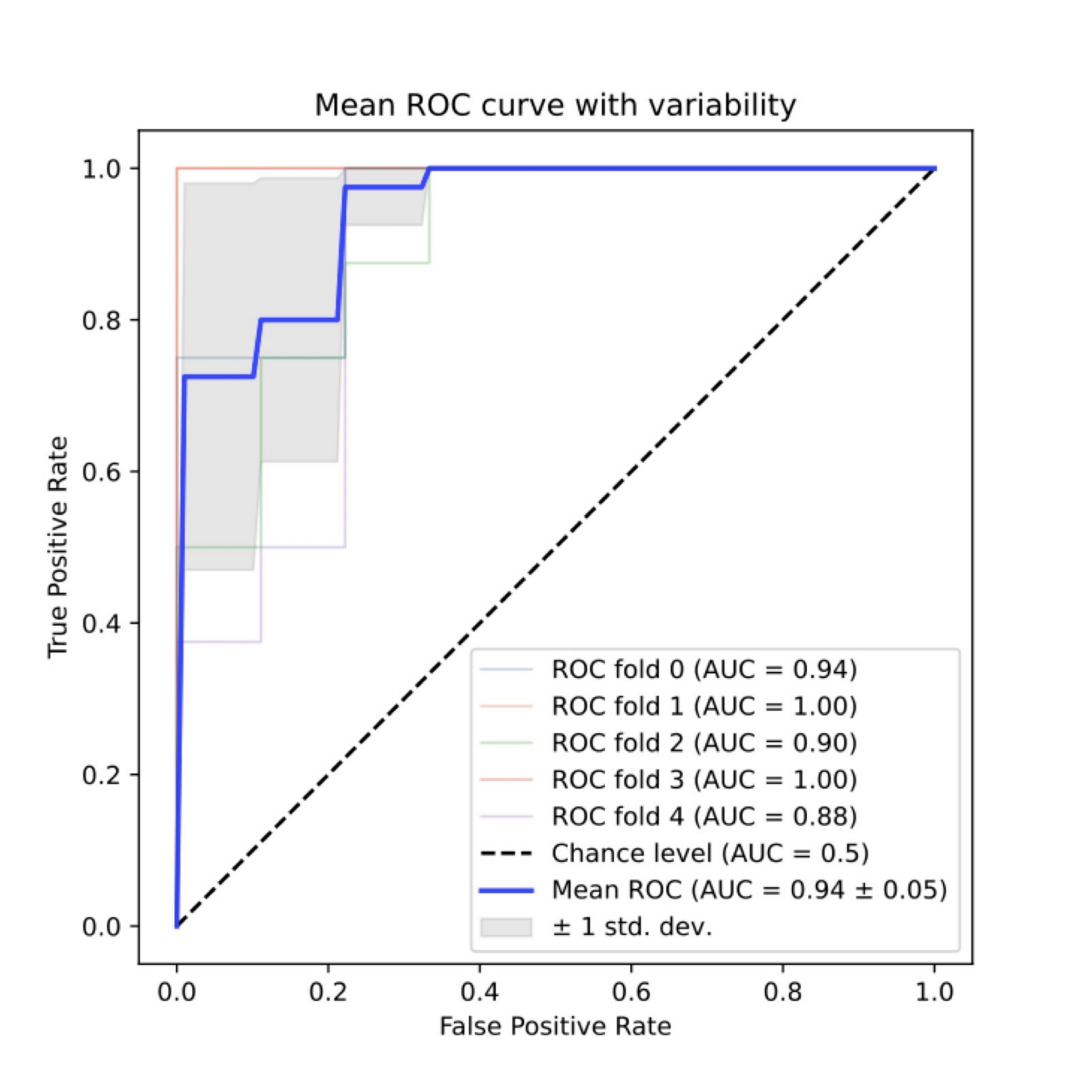
Receiver operating characteristic curve of highest performing predictive model for local response prediction of early-stage and oligometastatic patients, using wavelet-LHL filtered radiomics features. ROC, receiver operating curve; AUC, area under the curve; STD DEV, standard deviation

After filters were applied to the radiomic features, the highest-performing local response model, including all patients, improved from a mean AUC of 0.87 ± 0.09 to 0.94 ± 0.05, specifically with the wavelet-LHL filtered features (Figure 1).

**Figure 2:**
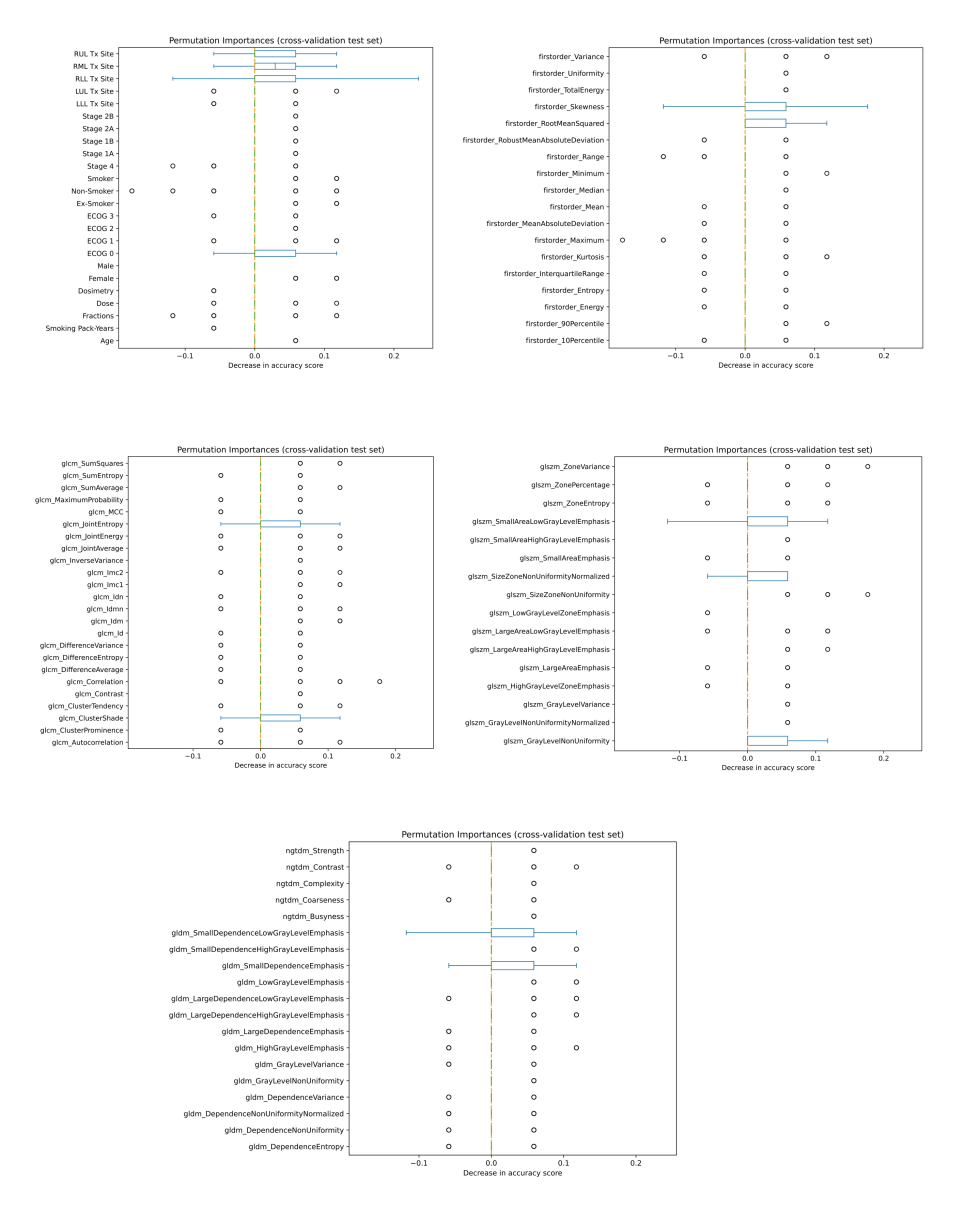
Permutation feature importance of highest performing predictive model for local response prediction of early-stage and oligometastatic patients. RUL, right upper lobe; RML, right middle lobe; RLL, right lower lobe; LUL, left upper lobe; LLL, left lower lobe; Tx, treatment; ECOG, Eastern Cooperative Oncology Group performance status; GLCM, gray-level co-occurrence matrix; GLSZM, gray-level size zone matrix; NGTDM, neighboring gray-tone difference matrix; GLDM, gray-level dependence matrix

The boxplots shown in Figure 2 illustrate the most important features involved in the combined-based, MLP model, with the ADASYN sampling method and the wavelet-LHL filter, when predicting local response in early-stage and oligometastatic patients. The treatment site and the performance score emerged as important, along with first-order features, such as skewness and root-mean-squared intensity. GLCM features included joint entropy and cluster shade. GLSZM features included small-area-low-gray-level emphasis, size-zone-non-uniformity normalized, and gray-level non-uniformity. GLDM features included small-dependence-low-gray-level-emphasis and small-dependence emphasis.

Table 3 compares performances between models but only for early-stage patients. While the random forest model was the highest performing, radiomics-based model (AUC = 0.92 ± 0.06), the SVC RBF model was both the best clinical-based and combined-based model with AUCs of 0.95 ± 0.06 and 0.93 ± 0.06, respectively. No statistical difference was found between these models (*P* = 0.73).

**Table 3:** Performance comparison of different models for local response prediction of only early-stage patients. ANOVA, analysis of variance; AUC, area under the curve; SD, standard deviation; ADASYN, adaptive synthetic; SVC, support vector classification; RBF, radial basis function; AdaBoost, adaptive boosting; MLP, multi-layer perceptron

Classifier	Data augmentation	Data input						One-way ANOVA
		Radiomics		Clinical		Combined		
		Mean (AUC)	SD (AUC)	Mean (AUC)	SD (AUC)	Mean (AUC)	SD (AUC)	*P*-value (alpha = 0.05)
SVC RBF, c1, ovo	No ADASYN	0.54	0.21	0.51	0.06	0.34	0.15	0.12
	ADASYN	0.86	0.1	0.95	0.06	0.93	0.06	0.19
SVC linear	No ADASYN	0.56	0.08	0.24	0.1	0.72	0.2	0.00042
	ADASYN	0.78	0.14	0.47	0.12	0.6	0.18	0.020
Random Forest Classifier, 55 estimators	No ADASYN	0.36	0.09	0.43	0.19	0.43	0.09	0.64
	ADASYN	0.92	0.06	0.84	0.1	0.83	0.12	0.31
AdaBoost, 300 estimators	No ADASYN	0.59	0.26	0.44	0.18	0.53	0.29	0.64
	ADASYN	0.87	0.09	0.74	0.06	0.83	0.1	0.084
MLP, sgd, alpha 0.001	No ADASYN	0.63	0.28	0.46	0.24	0.51	0.12	0.49
	ADASYN	0.87	0.13	0.77	0.05	0.91	0.07	0.076
ANOVA *P*-value comparison between best models		0.92	0.06	0.95	0.06	0.93	0.06	0.73

**Figure 3:**
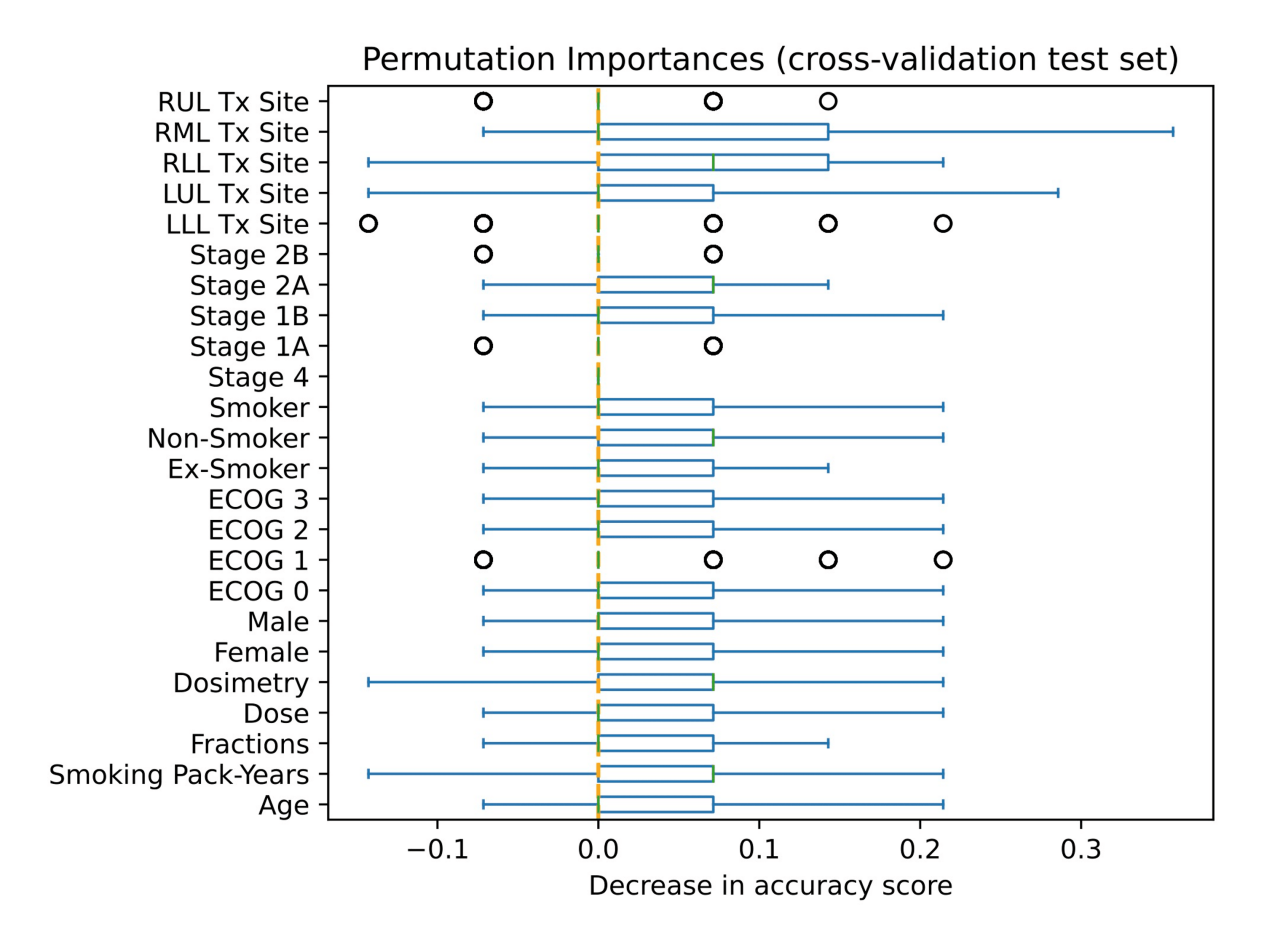
Permutation feature importance of highest performing predictive model for local response prediction of only early-stage patients. RUL, right upper lobe; RML, right middle lobe; RLL, right lower lobe; LUL, left upper lobe; LLL, left lower lobe; Tx, treatment; ECOG, Eastern Cooperative Oncology Group performance status

For the prediction of local response in only early-stage patients, the most important features in the clinical-based, SVC RBF model with the ADASYN sampling method were age, smoking pack-years, fractions, dose, dosimetry criteria (V12), gender, performance status, smoking status, and staging, with special emphasis on treatment site (Figure 3).

Among the patient dataset, the median age was 76 years, with a range going from 35 to 94 years and a male-to-female ratio of 46:52. As for survival, 29 patients died and 69 patients were lost to follow-up. The median survival after the first SBRT treatment was 36 months (three years), ranging from 1 month to 138 months. Also, the dataset was composed of 82 early-stage patients and 16 oligometastatic patients (Table 4). 

**Table 4:** Overall cohort demographics. Min, minimum; Max, maximum; SBRT, stereotactic body radiation therapy

Characteristics	Total	Average	Standard deviation	Median	Range (Min)	Range (Max)
Population	98	-	-	-	-	-
Age (Years)	-	75	9.8	76	35	94
Sex (Male)	46	-	-	-	-	-
Sex (Female)	52	-	-	-	-	-
Patients deceased	29	-	-	-	-	-
Patients lost to follow-up	69	-	-	-	-	-
Number of months between the start of SBRT and death/last follow-up (Months)	-	45	31	36	1	138
Patients with one Lesion	84	-	-	-	-	-
Patients with two Lesions	11	-	-	-	-	-
Patients with three Lesions	2	-	-	-	-	-
Patients with four Lesions	1	-	-	-	-	-
Staging at First SBRT - 1A	58	-	-	-	-	-
Staging at First SBRT - 1B	12	-	-	-	-	-
Staging at First SBRT - 2A	4	-	-	-	-	-
Staging at First SBRT - 2B	8	-	-	-	-	-
Staging at First SBRT - 4	16	-	-	-	-	-

There were 92 early-stage lesions and 24 oligometastatic lesions. When assessing the local response of all patients, 28% of patients had a positive response and 72% of patients had a negative response (Table 5). 

**Table 5:** Overall cohort treatment responses.

	Total	Positive response	Negative response
Number of lesions	116	-	-
Complete response (CR)	4	-	-
Partial response (PR)	29	-	-
Stable disease (SD)	58	-	-
Progressive disease (PD)	25	-	-
Local response (number of lesions, proportions (%)*	-	33 (28)	83 (72)

There were 92 early-stage lesions. After removing oligometastatic patients, the proportion of positive and negative responses remained relatively stable. Indeed, local response had a 25% positive response and a 75% negative response (Table 6). 

**Table 6:** Early-stage-only patients cohort treatment responses.

	Total	Positive response	Negative response
Number of lesions	92	-	-
Complete response (CR)	2	-	-
Partial response (PR)	21	-	-
Stable disease (SD)	48	-	-
Progressive disease (PD)	21	-	-
Local response, number of lesions (%)*	-	23 (25)	69 (75)

## Discussion

Local response

For early-stage NSCLC patients who refuse surgery or are medically inoperable, as well as for oligometastatic NSCLC patients, SBRT is established as the standard of care for the local control of early-stage primary and oligometastatic lesions located in the lung [4-6]. Not only is it a precise stereotactic method that reduces the exposure of surrounding healthy lung tissue to unnecessary radiation, but it also has a high rate of local control. However, there are cases where SBRT is not as effective as planned. It would be useful to have biomarkers capable of predicting early, local failure, by simply using pretreatment information, such as clinical data or radiomics data from the planning CT scan. In this study, to assess local response, CR and PR were considered as positive responses and SD and PD were considered as negative responses.

If we examine the performance of our radiomics-based model, the MLP model with the ADASYN sampling method achieved an AUC of 0.81 ± 0.1 (Table 1). This model echoes a similar but higher performance model from Cheung et al.'s study [26]. Indeed, when investigating the CT-based radiomic models that predicted local response for SBRT-treated, early-stage, and oligometastatic NSCLC, they obtained an SVM model with an AUC of 0.857 [26]. While Cheung et al.'s study [26] stopped at radiomics-based models, our study explored a multimodality approach, to potentially improve our predictive model’s performance.

Recently, Luo et al.'s study [27] developed a local response predictive model for SBRT-treated, stages I-II-III-IV, lung cancer patients (not specifically NSCLC patients), using a multimodality approach similar to our study [27]. Their best model used a logistic regression classifier and a combined-based input (their validation group had an AUC of 0.818), but the model was not trained with cross-validation [27]. In our study, the results presented in Table 1 and Table 2 demonstrate that, for early-stage and oligometastatic patients, the best model used a combined input with an MLP classifier, ADASYN oversampling, and wavelet-LHL-filtered radiomic features (AUC = 0.94 ± 0.05) (Figure 1). Thus, our model had more optimal training, and it was more performant than Luo et al.'s model [27]. Furthermore, similar to our study, Luo et al.'s study [27] employed a clinical and radiomic multimodality approach [27]. While their combined input model resulted in the best model, it was not statistically superior to their best radiomics-based and clinical-based models [27]. In our case, our combined-based model’s performance difference with our best radiomics-based and clinical-based models showed a trend towards superiority, but the difference was not statistically significant either (*P *= 0.063) (Table 2). Our study agrees with the observations of Luo et al. [27]; the combination of radiomics and clinical variables generally improves model performance, but it has not been proven to be statistically superior. This trend concurs with past studies demonstrating the value of a multivariate input when predicting local response in SBRT-treated lung cancer [28].

One objective of this project is to validate the stability of certain predictive biomarkers. This is especially important for radiomic features, which are known to be sensitive to various external factors [29,30]. Luo et al.'s study [27] determined that staging, platelet value, and the minimum biologically effective dose (BED) of the gross tumor volume (GTV) were significantly correlated to the prediction of local response [27]. Unfortunately, for their specific outcome prediction and specific cohort staging, our study did not validate Luo et al.'s biomarkers [27] because only the treatment site and the performance score emerged as predictive clinical features (Figure 2). On the other hand, we were able to validate the first-order radiomic biomarkers found in Cheung et al.'s study [26]. Indeed, when predicting the local response of SBRT-treated, early-stage, and oligometastatic NSCLC, similar to our study, they determined that first-order skewness and root-mean-squared intensity were important features [26]. Skewness and root-mean-squared-intensity were found to also be predictive of response in slightly different situations, such as chemo-irradiated tumors in different cohorts of lung cancer [31,32]. By validating the skewness and the root-mean-squared-intensity features, we are supporting their use as consistent CT-based biomarkers for local response prediction. Other important features found in our study included GLCM features (joint entropy and cluster shade), GLSZM features (small-area-low-gray-level emphasis, size-zone-non-uniformity normalized, and gray-level non-uniformity) and GLDM features (small-dependence-low-gray-level emphasis and small-dependence emphasis) (Figure 2).

Moreover, our study aimed to illustrate the effects of cohort specificity on a predictive model’s performance. To accomplish this goal, we trained models that included both early-stage and oligometastatic patients, as well as models that focused solely on early-stage patients. In Lafata et al.'s study [12], they used only radiomic features to predict local control for stage I, SBRT-treated NSCLC tumors, generating a logistic regression model with an AUC of 0.83 ± 0.03 [12]. Similarly, in Avanzo et al.’s study [33], they attempted to improve machine learning models that could predict local response for early-stage NSCLC treated with SBRT [33]. They used an ensemble machine learning (EML) method with robust boosting as their classifier, and they trained their model with combined radiomics and BED input. This model resulted in an AUC of 0.773 [33]. In comparison, using stage I and stage II NSCLC patients and only clinical features, our study generated an SVC RBF model with a higher AUC of 0.95 ± 0.06 (Table 3). Unlike our study and Avanzo et al.’s study [33], Lafata et al.’s study [12] did not use clinical input when training their models [12]. When comparing Lafata et al.’s best models [12] and this study, clinical features were in general more predictive than radiomic features, when predicting local response in early-stage patients. Avanzo et al.'s study [33] reinforces this trend, as they found that using BED with CT radiomics resulted in a significantly higher AUC compared to models based exclusively on CT radiomics [33]. In our study, a one-way ANOVA showed no statistical difference between the best radiomics, clinical, and combined models (*P* = 0.73) (Table 3). If we had corrected the dosimetry parameter (V12 for lung) from our clinical data input to a biologically effective dose [33], the performance of our clinical-based and combined models might have been statistically superior to that of our radiomics-based model.

As for important predictive biomarkers, Lafata et al.'s study [12] found that homogeneity and long-run high-gray-level emphasis were significantly important in predicting local control, using univariate feature analysis [12]. Among the features included in Avanzo et al.’s data inputs [33] and ours, Avanzo et al.’s study [33] found that the most important features were GLCM cluster prominence, GLRLM large-zone-(area)-high-gray-level emphasis, and GLSZM large-zone-(area)-high-gray-level emphasis [33]. Unfortunately, our study was not able to validate Lafata et al.’s [12] and Avanzo et al.’s [33] features because of the scope and design of this study. We only conducted permutation feature importance on the best models; however, in this case, the best models only used clinical features. In our study, the feature found as the most predictive of local response in early-stage patients was the treatment site (Figure 3).

Limitations

The retrospective nature of this study introduced limitations. For instance, there was a risk of introducing selection bias in our dataset. An independent, external, validation dataset is required to evaluate any potential selection bias. Furthermore, patients involved in a retrospective study carry various uncontrolled variables; then, it becomes more difficult to conclusively associate a treatment/intervention to an outcome, compared to prospective studies. Also, as our retrospective study relied on past, medical records, some patient data were not available. As many physicians and residents from 2009 to 2022 originally wrote our patients’ charts, the data collected from one patient to the next did not have the same extent of details, thus limiting the type of variables collected for our study. Even then, we had to use data imputation to replace missing data.

Inter-observer uncertainty was another limitation impacting multiple aspects of our study. For example, when assessing local tumor response using RECIST, there is up to 24% uncertainty when evaluating the change in the sum of the longest diameters of tumors from the pre-treatment CT scan to the last follow-up CT scan [34]. Furthermore, the lack of a universal, feature extraction protocol could potentially limit inter-institutional reproducibility. While our study followed the TRIPOD statement by extracting radiomic features using PyRadiomics, there are many radiomic feature extraction modules and software, spanning different coding platforms [30].

Potential advancements and future research

While this study generated interesting insights about the use of predictive algorithms for SBRT, further research should be conducted to develop a deeper understanding of clinical and radiomic features and further validate our findings.

For the validation of our models in a multi-institutional setting, establishing an official, working pipeline between institutions would be critical to advance our project. For this collaboration to work, a multi-institutional contract should be established to ensure that researchers involved in our project would easily access the anonymized data and scans needed for external validation.

By collaborating with other institutions, we could even expand the interpretation of our predictive tools to slightly different situations. For example, in Yang et al.’s prospective exploratory clinical study (MISSILE-NSCLC, NCT02136355) [35], they developed a model for the prediction of pathological complete response (pCR) for early-stage patients treated with SBRT and surgery, using imaging-based biomarkers from dynamic fludeoxyglucose-18-PET (FDG-PET) and CT perfusion [35]. We could advance both our study and theirs by collaborating with them to conduct a prospective phase II trial involving early-stage NSCLC patients who would undergo SBRT followed by surgery. We would use our models, along with a combined version that includes their biomarkers, for the predictive assessment of pCR to SBRT. Predictions would be made before SBRT is administered and compared to follow-up assessments of disease progression. Assessing the capabilities of our models to predict pCR versus no pCR would be of key interest.

Finally, to fully understand important features and use them in a clinical setting, further research could be conducted to establish the biological mechanisms behind the biomarkers. For instance, first-order skewness, identified as a predictive radiomic feature in the stage I-II-IV local response model, is associated with poor radiotherapy response and radioresistance in lung cancer and other cancer types [36-38]. Radiogenomics studies found that positive skewness in NSCLC tumors is associated with KRAS mutations [39]. Even then, further research is required to verify if the radiologically observed skewness is responsible for poor local control by affecting the tumor density and texture or if skewness is only a biomarker of KRAS mutations.

## Conclusions

The predictive models created and the biomarkers identified/validated could be used in clinical decision support systems. Clinicians would greatly benefit from having predictive characteristics and algorithms, which could foreshadow a patient’s response to radiation therapy. Indeed, these tools could spare the minority of patients who do not benefit from SBRT, thus helping physicians to adjust their patients’ treatment course. Using these models and biomarkers in prospective trials is crucial to evaluate the clinical utility. These advances in treatment decision systems could shift the current, medical practice from a systematic decision-making process to personalized medicine. By refocusing healthcare on the unique characteristics of patients, we could optimize patient care, ensuring that every treatment is both beneficial and impactful.
